# Automatic detection and vascular territory classification of hyperacute staged ischemic stroke on diffusion weighted image using convolutional neural networks

**DOI:** 10.1038/s41598-023-27621-4

**Published:** 2023-01-09

**Authors:** Kun-Yu Lee, Chia-Chuan Liu, David Yen-Ting Chen, Chi-Lun Weng, Hung-Wen Chiu, Chen-Hua Chiang

**Affiliations:** 1 Department of Medical Image, Shuang Ho Hospital, Taipei Medical University, No. 291, Zhongzheng Road, Zhonghe District, New Taipei City, 23561 Taiwan, ROC; 2 Department of Radiology, School of Medicine, College of Medicine, Taipei Medical University, No. 250 Wu-Hsing Street, Taipei City, 11031 Taiwan, ROC; 3 Department of Radiology, Ditmanson Medical Foundation Chia-Yi Christian Hospital, No. 539, Zhongxiao Rd., East Dist., Chiayi City, 600566 Taiwan, ROC; 4 Graduate Institute of Biomedical Informatics, College of Medical Science and Technology, Taipei Medical University TW, No. 250 Wu-Hsing Street, Taipei City, 11031 Taiwan, ROC

**Keywords:** Neuroscience, Medical research, Neurology, Computer science

## Abstract

Automated ischemic stroke detection and classification according to its vascular territory is an essential step in stroke image evaluation, especially at hyperacute stage where mechanical thrombectomy may improve patients’ outcome. This study aimed to evaluate the performance of various convolutional neural network (CNN) models on hyperacute staged diffusion-weighted images (DWI) for detection of ischemic stroke and classification into anterior circulation infarct (ACI), posterior circulation infarct (PCI) and normal image slices. In this retrospective study, 253 cases of hyperacute staged DWI were identified, downloaded and reviewed. After exclusion, DWI from 127 cases were used and we created a dataset containing total of 2119 image slices, and separates it into three groups, namely ACI (618 slices), PCI (149 slices) and normal (1352 slices). Two transfer learning based CNN models, namely Inception-v3, EfficientNet-b0 and one self-derived modified LeNet model were used. The performance of the models was evaluated and activation maps using gradient-weighted class activation mapping (Grad-Cam) technique were made. Inception-v3 had the best overall accuracy (86.3%), weighted F1 score (86.2%) and kappa score (0.715), followed by the modified LeNet (85.2% accuracy, 84.7% weighted F1 score and 0.693 kappa score). The EfficientNet-b0 had the poorest performance of 83.6% accuracy, 83% weighted F1 score and 0.662 kappa score. The activation map showed that one possible explanation for misclassification is due to susceptibility artifact. A sufficiently high performance can be achieved by using CNN model to detect ischemic stroke on hyperacute staged DWI and classify it according to vascular territory.

## Introduction

Ischemic stroke is a complex disease which can cause much patient disability or even mortality. It is the second most common cause of death worldwide and the third most common cause of disability after neonatal conditions and ischemic heart disease^1^. It is also one of the top three causes that resulted in premature mortality^2^. The absolute number of people affected by stroke has substantially increased across all countries in the world from 1990 to 2013, suggesting that the global stroke burden continues to increase^3^.

Ischemic stroke is a highly time-dependent disease. It can be classified and dated according to its age as early hyperacute (0–6 h), late hyperacute (6–24 h), acute (24 h to 7 days), and chronic (more than 3 weeks)^4^. The phrase “time is brain” emphasizes that human nervous tissue is rapidly lost as stroke progress and emergent evaluation and therapy are required^5^. Compared with the normal rate of neuron loss in brain aging, the ischemic brain ages 3.6 years each hour without treatment^5^. Thus, the current guideline suggests that patients with hyperacute stroke should receive thrombolytic and neurointerventional therapies^6^, which requires rapid image evaluation and vascular territory classification.

Ischemic stroke in different vascular territories usually presents with specific symptoms and signs. For example, anterior circulation infarct can produce motor and/or sensory deficits, and posterior circulation infarct can cause symptoms such as limb and gait ataxia, dizziness, nausea, vomiting, or even coma. Neurologists rely on these symptoms and signs to classify and localize which vascular territory is potentially affected by the infarction.

However, due to the complexity of our cerebral anatomy, ischemia in different parts of the brain can produce similar neurological signs and symptoms. Tao et al. evaluated the frequency of symptoms/signs in the 2 vascular territories to determine the diagnostic value of particular symptoms/signs for posterior circulation infarct (PCI) and found that the symptoms/signs considered typical of PCI occur far less often than was expected^7^. Therefore, inaccurate localization would occur commonly if clinicians relied on the clinical neurological deficits alone to differentiate PCI from anterior circulation infarct (ACI). As a result, neuroimaging is vital to ensure accurate localization of cerebral infarction^7^. Amongst computed tomography (CT) and many different sequences of magnetic resonance imaging (MRI), diffusion-weighted imaging (DWI) of MRI is considered to be the gold standard for the sensitive detection and diagnosis of acute ischemic stroke and is relied heavily on the clinical setting for detection and localization of ischemic stroke^8^.

Deep learning refers to neural networks with many layers (usually more than five) that extract a hierarchy of features from raw input images^9^. It is a form of supervised machine learning method that uses some form of neural network. Compared with traditional machine learning methods, deep learning can extract a complex hierarchy of features from images due to their self-learning ability as opposed to the hand-crafted feature extraction in classical machine learning algorithms which require human expertise^10^. More importantly, deep learning has been shown to achieve impressive results and generalizability if training on a large data.

There are many types of deep learning methods that have been developed over the years for different purposes. Among them, the convolutional neural network (CNN) is the most common and successful algorithm used in the computer vision task. CNN has been shown to adapt very well to computer vision. Many applications or algorithms have been developed or researched on medical imaging in the areas of image transformation, lesion detection, segmentation, and even image-based diagnosis^11^.

With these recent advances in deep learning, we hope to automate the process of stroke image analysis, which requires experts’ knowledge and experience and is time-consuming. In this study, we aimed to automate the process of classifying acute ischemic stroke into ACI, PCI, or normal images using DWI by CNN models, so that the clinician and other medical personnel can respond to patients with acute ischemic stroke more efficiently and increase the chances for patients to receive early treatment.

## Materials and methods

### Institutional review board

This retrospective study was approved by Taipei Medical University—Joint Institutional Review Board (No. N201912116). This study was conducted in accordance with relevant local guidelines and regulations and the requirement for informed consent was waived.

### Study population

We searched the MRI examination list in our department to identify the number of patients who had the examination type ‘MRI for IA’, which was the examination protocol for patients presented with suspicious hyperacute infarct from January 2018 to November 2019 and may benefit from intra-arterial mechanical thrombectomy.

Using this method, we identified 127 patients in the year 2018 and 126 patients in the year 2019. We enrolled these patients retrospectively and downloaded their DWI in Digital Imaging and Communications in Medicine (DICOM) format. We excluded those patients who had no image evidence of infarction, who presented with infarctions other than anterior and posterior infarction (for example, patients who had lacunar infarct, watershed infarct) and who suffered from severe motion or other artifacts and we ended up with a total of 127 cases. The age and gender of these patients were recorded.

Of the 127 patients, 60 (47%) were male and 67 (53%) were female. The mean age in our studied population was 70.2, and the youngest age was 42 and the oldest age was 98. All the demographic data are shown in Table 1.

**Table 1:** Gender and age distribution of the cases.

	Male	Female	Total
ACI	48 (47.5%)	53 (52.5%)	101 (100%)
PCI	12 (46.2%)	14 (53.8%)	26 (100%)
Total	60 (47.2%)	67 (52.8%)	127 (100%)
Mean age (interval)	71.2	69.8	70.2 (42–98)

### Image acquisition

MR images were performed using two machines, including 1.5 T (Signa HDX, GE Healthcare, Milwaukee, WI, USA) and 3 T scanners (Discovery MR750, GE Healthcare, Milwaukee, WI, USA). The parameters for the DWI sequences included the following: repetition time = 8000 ms; echo time = minimum; image matrix size = 256 × 256; spacing between slices = 2 mm; number of slices = 20–24; slice thickness = 5 mm; b-value = 1000 s/mm^2^. FOV for both scanners was 230. Matrix for to 3 T scanner was 128 × 128 whereas for the 1.5 T scanner was 120 × 160. Pixel size for the 3 T scanner is set to be 1.8 × 1.8 mm and for the 1.5 T scanner was 1.9 × 1.4 mm.

### Patient dataset

We then converted these DW images into PNG (Portable network graphic) format and separated them into three file folders, namely: anterior circulation infarct, posterior circulation infarct, and normal (Table 2). We excluded images that were at the extreme end of the skull base and top of the skull or images which did not include the brain as these images contained no information of infarction nor normal brain anatomy. Two radiologists each with 10 years and 6 years of experience (C.H.C and K.Y.L respectively) were responsible for the ground-truth creation. Both radiologists reviewed the images and classified these images into three classes individually and in the cases that had difficulties in classification, the consensus was reached between the two radiologists.

**Table 2:** Dataset distribution.

	Anterior circulation infarct	Posterior circulation infarct	Normal	Total
Number of images (percentage)	618 (29.2)	149 (7.0%)	1352 (63.8)	2119

### Designing, training and validation of our CNN architecture

After compiling 2119 images from 127 patients, we randomly assigned images for training and validation at a ratio of 7:3. We first used our self-designed CNN architecture, which was modified from the simple “LeNet” structure, and this CNN architecture has been published for automatic medical image location and modality classification previously^12^. LeNet was one of the earliest CNNs proposed by LeCun et al. in 1998 and it is a form of multilayer neural networks trained with the backpropagation algorithm. It was applied to handwritten character recognition and shown to outperformed traditional methods at that time^13^. We changed the setting of maximum epochs into 40 epochs and initial learning rate of 0.001. We also adjusted to random shuffling after each epoch and minibatch size of 32.

### Transfer learning using deep CNN models

In addition to our own proposed CNN architecture, two different pre-trained CNN models were also trained and validated in this study, namely, Inception-v3 and EfficientNet-b0 architectures for the classification of infarct location. Inception-v3 is a modification of GoogLeNet, which uses the structure of inception^14^. The main hallmark of the inception structure is that it improved utilization of the computing resources inside the network through multi-scale processing^15^. Inception-v3 has been previously investigated in stroke image assessment on both DW image^16^ and CT image^17^ with good results. EfficientNet-b0 is known to uniformly scale all dimensions of depth/width/resolution which allows it to achieve much better accuracy and efficiency than previous CNNs^18^. It has been previously used by Cetinoglu et al. for detection and vascular territorial classification on DW images and achieved good result^19^.

Both CNN architectures are well known models that were pretrained using ImageNet dataset, which had more than a million images and these CNN models can classify images into 1000 object categories and both had their success with ImageNet dataset.

As these pre-trained models accept different input shape dimension (Inception-v3: 299 × 299 × 3, EfficientNet-b0: 224 × 224 × 3), we created a new image input layer which accepted 256 × 256 × 1 shape and then an additional convolution layer of 1 × 1 × 3 to converted the images into 3 channels. We also replaced the original fully connected and classification layers for them to classify 3 classes instead of original 1000 classes in the ImageNet. These CNN models were all trained using stochastic Gradient Descent (SGD) with momentum optimizer. Initial learning rate were set to be 0.001, and mini-batch size of 32 images with 40 maximum epochs.

We performed all three of our models several times in order to fine-tune the hyperparameters. Each time, we randomly split our dataset into 7:3 (training: validation) ratios according to 3 categories. We recorded and reported the best validation result after our trials.

All these tasks were performed utilizing MATLAB 2021b running on a computer incorporated with Intel Core i7 2.7 GHz CPU, 16G main memory and Intel HD Graphics 530 1536 MB.

### Statistical analysis and generation of activation map

The performance of different networks was evaluated by creating confusion matrix to derive several performances metrics, namely accuracy, sensitivity, specificity, weighed F1 score and kappa score. In order to understand the reasons for misclassification, we used gradient-weighted class activation mapping (Grad-CAM) technique proposed by Selvaraju et al.^20^. Grad-CAM technique uses the feature maps produced by the last convolutional layer of a CNN and the gradients flowing into that final layer, then calculates an importance score based on these gradients. It can then identify the parts of an image that impacted the importance score the most and makes a coarse localization map that highlights the important regions in the input image for the CNN to make the prediction. This technique provides "visual explanations" to what regions of an image a model used for prediction and can be used to analyze reasons that a model fails to predict the desired result. It is also very versatile as it can produce visual explanations from any arbitrary CNN models. A schematic explanation of applying Grad-CAM on CNN architecture can be found on the original paper written by Selvaraju et al.^20^.

## Results

Of all 127 patients, 101 (79.5%) patients had ACI and 26 (20.5%) patients had PCI. After separating all the image slices into normal, ACI and PCI. The normal file contained 1352 image slice, ACI file contained 618 image slices and the PCI file contained 149 images. 81 cases (63.8%) were collected from 1.5 T MRI device and 46 cases (36.2%) was collected from 3 T MRI device.

**Figure 1:**
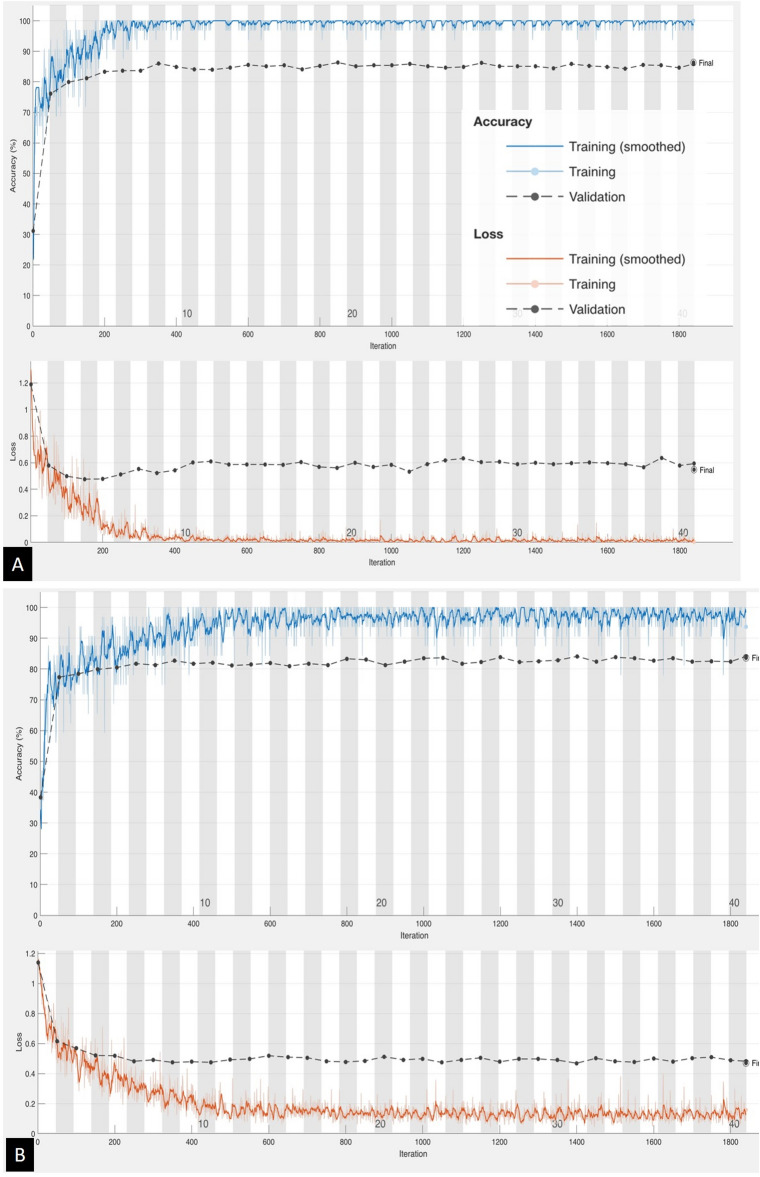

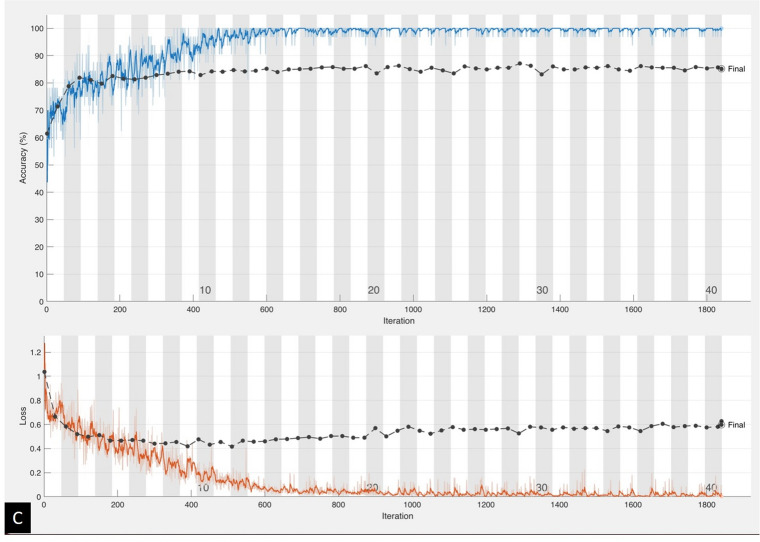
Accuracy and loss graphs of Inception-v3 model (**A**), EfficientNet-b0 model (**B**) and Modified LeNet model (**C**).

We used previously mentioned Inception-v3, EfficientNet-b0 and the modified LeNet CNN models for training and validation, the total time elapsed for the training and validation were 335, 410, and 35 min respectively. From 7 to 10th epoch, each of the CNN model learning curve started to stabilize. The best validation accuracy on the final epoch of Inception-v3, EfficientNet-b0 model and the modified LeNet CNN model was 86.3%, 83.6% and 85.2% respectively. The accuracy and loss graph of each CNN models are shown in Fig. 1. The confusion matrices and calculated metrics (accuracy, precision, sensitivity, specificity, weighted F1-score, total accuracy and kappa score) of all the models in detecting the presence of stroke are shown in and Tables 3, 4, 5 and 6.

**Table 3:** Confusion matrix of Inception-v3 model in classifying stroke vascular territory.

(Predicted value)	Inception-v3			
ACI	147	24	4	
Normal	37	380	19	
PCI	1	2	22	
	ACI	Normal	PCI	(Actual value)

**Table 4:** Confusion matrix of EfficientNet-b0 model in classifying stroke vascular territory.

(Predicted value)	Efficientnet-b0			
ACI	146	34	4	
Normal	39	367	22	
PCI	0	5	19	
	ACI	Normal	PCI	(Actual value)

**Table 5:** Confusion matrix of Modified LeNet model in classifying stroke vascular territory.

(Predicted value)	Modified lenet			
ACI	150	31	1	
Normal	35	372	24	
PCI	0	3	20	
	ACI	Normal	PCI	(Actual value)

**Table 6:** Calculated metrics of all the models in classifying stroke vascular territory.

	Inception-v3			Efficientnet-b0			Modified lenet		
	ACI	Normal	PCI	ACI	Normal	PCI	ACI	Normal	PCI
Accuracy	89.6%	87.1%	95.9%	87.8%	84.2%	95.1%	89.4%	85.3%	95.5%
Precision	84.0%	87.1%	88.0%	79.3%	85.7%	79.1%	82.4%	86.3%	86.9%
Sensitivity	79.4%	93.5%	48.8%	78.9%	90.3%	42.2%	81.0%	91.6%	44.4%
Specificity	93.7%	75.6%	99.4%	91.5%	73.4%	99.1%	92.9%	74.3%	99.4%
F1 score	81.9%	90.9%	63.0%	79.1%	88.2%	55.2%	81.9%	89.2%	58.8%
Overall accuracy		86.3%			83.6%			85.2%	
Weighted F1 score		86.2%			83.0%			84.7%	
Κ score		0.715			0.662			0.693	
Total time elapsed (min)		335			410			35	

**Figure 2:**
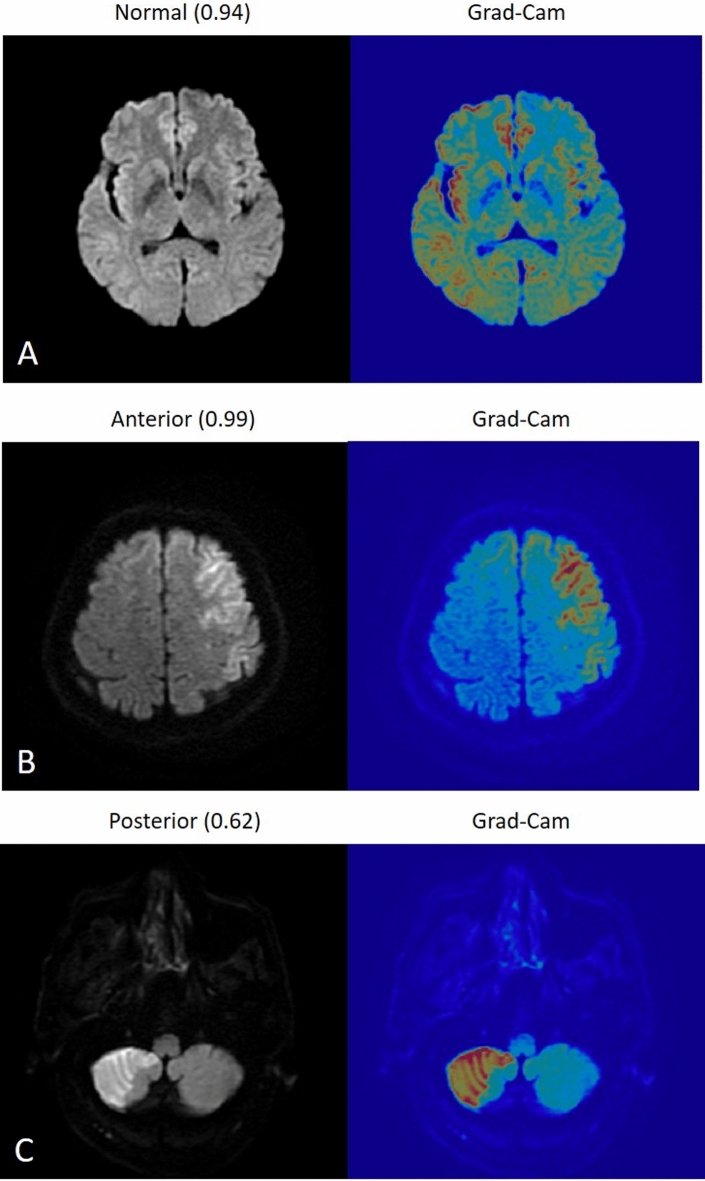
Example images that were correctly classified by the modified LeNet model, the first case was normal (**A**), the second one was left frontal and parietal infarction which was correctly classified into ACI (**B**), and the third one was right cerebellar infarction which was correctly classified in PCI (**C**). The number in bracket represent classification score.

**Figure 3:**
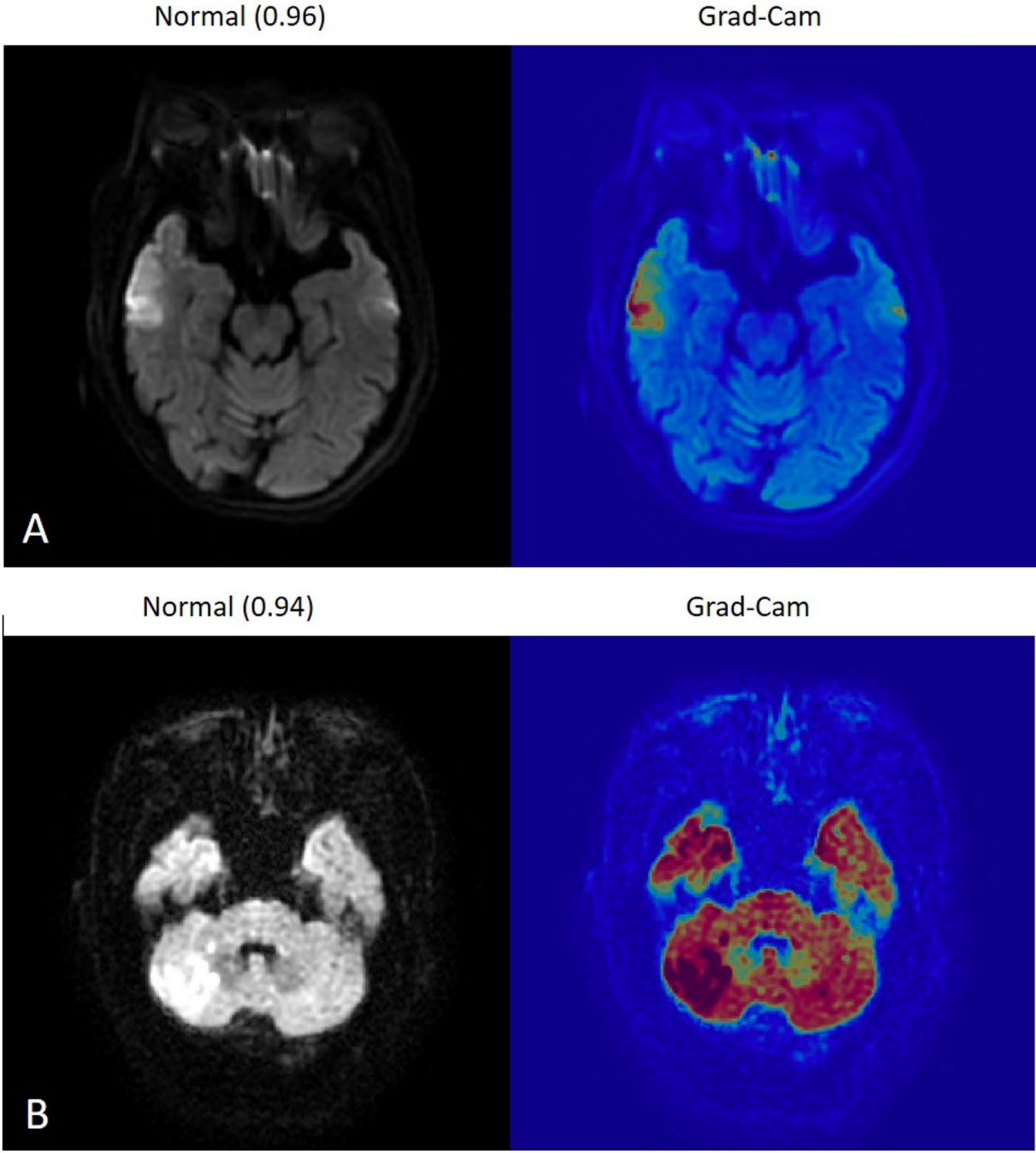
Example images of misclassification, the first case was right temporal lobe infarction, but miss-classified into normal by EfficientNet-b0 model (**A**), and second case was right cerebellar infarction but miss-classified into normal by the modified LeNet model (**B**). The number in bracket represent classification score.

The activation map generated using the Grad-CAM technique applied on the validation DW images and classification score is shown in the figure, regions where the color is more towards red, represent areas activated by the CNN. The dark purple background represents areas that were not activated. Figure 2 shows the examples of correct classification, the first case was a normal image, the second one was classified as ACI with left frontal region being activated and the third one was classified as PCI where right cerebellar was activated by the CNN model. Figure 3 shows two misclassified sample images. First case was an ACI case with infarction area at right temporal lobe, but CNN misclassified it into normal. The second case was a PCI case with an area of DWI bright up over right cerebellum but CNN misclassified it into normal.

## Discussion

Among the three models, the Inception-v3 model had the best accuracy, weighed F1 score and kappa coefficient, followed by our modified LeNet, and the EfficientNet-b0 had the poorest performance. It was rather surprising that our simple CNN, modified LeNet, outperformed the much more complex and deeper EfficientNet-b0 in this specific task. Compared with the Inception-v3 and EfficientNet-b0, our modified LeNet not only had very comparable performance, but it also took the least time to train and validate (35 min), making the modified LeNet the most efficient architectures among the three CNN models in this stroke location classification task.

The three CNNs all had the highest sensitivity in classifying normal images, followed by ACI image slices, and PCI showed the least sensitivity. The sensitivity of PCI classification ranged between 42.2 and 48.8% among the three networks. This may be explained by the fact that our dataset contained the greatest number of normal image slices, followed by image slices of ACI and then PCI. This order of sensitivity also affected the weighted F1 score, causing the normal image slices had the best F1 score amongst the three groups, followed by the ACI image slices and then the PCI image slices.

Our collection of image slices between anterior and posterior circulation showed an imbalanced distribution of data. Among a total number of image slices of infarction, 80.6% was of anterior circulation infarction and 19.4% was of posterior circulation infarction. As this data was originally collected from those patients who had hyperacute stage infarction that might benefit from mechanical thrombectomy, this distribution of our ACI and PCI slices was compatible with the one published by Chu et al.^21^, in which they collected cases that had performed endovascular thrombectomy between 2014 to 2016 and found 86.2% of cases were of anterior infarction and 13.8% was of posterior circulation infarction. Therefore, despite our imbalance of ACI and PCI image slices, our dataset still reflected the ratio of anterior and posterior circulation in clinical practice in Taiwan.

As far as we know, Cetinoglu et al. is the only one research paper that aimed to detect vascular territorial classification of stroke using DWI by CNN models^19^. They had two datasets, one dataset was for stroke detection purposes, and it contained 900 stroke slices and 900 slices of the normal brain, and another vascular territorial dataset was created with 1717 slices and grouped into MCA stroke, PCI, and watershed stroke categories. They used a transfer learning approach based on MobileNetV2 and Efficient Net-B0 CNN architecture and were able to achieve above 90% stroke detection rate and above 85% accuracy for vascular territorial classification. In our study, we had only one dataset which contained hyperacute staged DWI of both stroke and normal and we aimed to classify these images into anterior, posterior circulation territorial infarct and images without infarction, which required the model to perform both detections of stroke and classification into vascular territory at the same time, therefore it is a more difficult task compared with prior study. What is more, our image selection of hyperacute staged DWI is known to present heterogeneous and patchy hyperintense pattern, which could sometimes even pose difficulties for human expert readers^22^. A combination of these factors can therefore explain our models’ poorer performance compared with the work done by Cetinoglu et al.

In our study, we used three different CNN models for a combination of ischemic stroke detection and classification of hyperacute ACI and PCI (< 24 h), which was potentially eligible cases for mechanical thrombectomy. Despite the fact that there are several differences in the presenting symptoms such as patients with PCI usually present with decreased consciousness, visual field defects, and vestibulo-cerebellar signs, but fewer hemisyndromes, dysarthria, and cognitive symptoms compare with patients presented with ACI^23^. More than one-third of PCI are misdiagnosed in emergency department, more than three times as ACI^24^. Mechanical thrombectomy in ACI and PCI also has different characteristics, for example, mechanical thrombectomy in PCI has a lower risk of symptomatic intracranial hemorrhage compared to ACI, and patients with PCI seemed to benefit from MT started beyond 6 h after symptom onset^25^.

Current commercial software on stroke image focus on calculating ischemic stroke core and penumbra volume and require physicians to determine the involved vascular territory, despite the many difference in presenting symptoms and signs of vascular territory and post-thrombectomy outcomes. Therefore, this method of automatic classification of ACI, PCI, and normal images may be a useful first step of automatic software to first classify infarction location and then calculate the volume of infarct according to the respective infarct territory. Rapid diagnosis and territorial classification are important for initiation of thrombolytic therapy and reduction of door-to-puncture time in mechanical thrombectomy, which may significantly improve the clinical outcome in hyperacute stroke patients^26^.

Using Grad-CAM, we discovered that one potential explanation for the reasons for misclassification may be due to susceptibility artifact, which is a common finding at the air-bone interface of the temporal bone in DWI^27,28^. This artifact frequently creates patchy bright signal along bilateral petrous apex with spatial distortion on DWI which may have misled our training model so that it took the right temporal bright up region as an artifact in the first case of Fig. 3 and resulted in the misclassification. This susceptibility artifact occurs frequently in the clinical setting and can sometimes be difficult even for human readers to differentiate whether the DWI bright-up region is due to an artifact or representing true ischemic infarction.

Our study has several limitations. The first limitation was that no test set was created to test the true performance of our CNN models. The reason was that our dataset suffers from data imbalance, especially in the PCI group. We therefore repeated our training and validation several times for each model with random sampling as an attempt to reduce the bias. The second limitation was that we only used DW images for training and validation, if we incorporated apparent diffusion coefficient (ADC) images which simulates how human radiologist detect ischemic infarction, we might achieve a better result. The third limitation was the limited type of data diversity. Although we had two MR machines in our center, our CNN models only took images from a single center with the MR machines made by the same vendor, which may not be generalized enough to achieve a similar result in other image parameters or MRI machines. Therefore, a need to test the performance of our CNN models on multi-institutional data is needed. The fourth limitation was that all of the normal image slices were collected from patients who suffered from stroke, which may compromise the validation. It would have been more suitable to use normal MR images of non-stroke healthy patients in the normal class.

## Conclusion

Detection of hyperacute staged ischemic stroke and classification according to vascular territory is the first step in stroke image evaluation. It is also vital in pre-thrombectomy image evaluation as ACI or PCI has different management plans and carries different risks and potential outcomes. However, this step of image evaluation requires human expertise, is time-consuming, and currently no stroke image commercial software that we know of incorporates this in the automated process.

Our study showed that using either transfer learning-based CNN models or a self-modified simple CNN model, we achieved an at least above 83% accuracy rate and reached a substantial agreement of kappa score in our task of both stroke detection and territorial classification using hyperacute staged DW images. This would be an important addition to the currently available commercial software. Further external validation would be needed to assess our models’ generalizability.

## Data Availability

The data are not publicly available due to institution ethical and legal restrictions imposed by the Ethics Committee of Taipei Medical University, but will be made available on reasonable request and permissions from the corresponding author.
